# The Bamboo Flowering Cycle Sheds Light on Flowering Diversity

**DOI:** 10.3389/fpls.2020.00381

**Published:** 2020-04-17

**Authors:** Xiao Zheng, Shuyan Lin, Huajun Fu, Yawen Wan, Yulong Ding

**Affiliations:** ^1^Co-Innovation Center for Sustainable Forestry in Southern China, Nanjing Forestry University, Nanjing, China; ^2^Bamboo Research Institute, Nanjing Forestry University, Nanjing, China; ^3^College of Biology and the Environment, Nanjing Forestry University, Nanjing, China

**Keywords:** *Bambusoideae*, bamboo flowering events, theory of bamboo flowering cycle, rejuvenation, flowering diversity

## Abstract

Bamboo is a perennial flowering plant with a distinctive life cycle: many bamboo species remain in the vegetative phase for decades, followed by mass synchronous flowering and subsequent death. The phenomenon of bamboo flowering is not fully understood, but its periodicity is a major research focus. Here, we collected information on bamboo flowering events by investigating historical documents and field studies at the Bamboo Research Institute of Nanjing Forestry University. We compiled information on more than 630 flowering events, 124 of which accurately recorded the flowering cycle time. We summarized the specific flowering cycles of 85 bamboo species, as well as four kinds of bamboo flowering habits in detail. We present a theory of the bamboo flowering cycle and discuss the reasons for the observed variations in bamboo flowering. This review also introduces two mechanisms by which bamboo forests are rejuvenated after flowering and explains the flowering phenomena of bamboo forests using the bamboo flowering cycle theory. Finally, we present suggestions for forest management strategies. Bamboo flowering is a normal physiological phenomenon, even though it has unique elements compared with flowering in other plants. The results presented here provide valuable reference material for understanding bamboo flowering and its periodicity.

## Introduction

Bamboo (subfamily Bambusoideae) is a large clade of the family Poaceae. There are more than 88 genera and 1,642 species worldwide, of which 28 genera and more than 120 species are herbaceous bamboo (Vorontsova et al., 2016). Bamboo is widely distributed in the tropical, subtropical, and temperate regions of all continents, except Europe and Antarctica, from lowlands to ~4,000 m above sea level. China is a center of bamboo distribution, having more than 34 genera and 534 species (Chen et al., 2006). Bamboo species play important economic and ecological roles in many countries. Bamboo provides a wide range of products and has many uses for humans and other animals, while also having a major impact on the environment (Zhou, 1984).

Bamboo has attracted worldwide attention because of its distinctive life history. It is a perennial flowering plant, but many bamboo species remain in a vegetative phase for decades, or even a century, followed by mass synchronous flowering and subsequent death (Janzen, 1976). Thus, bamboo flowering has a negative effect on the livelihoods of people who depend on bamboo resources and could lead to famine among self-sufficient farmers. For example, *Bambusa balcoa, B. tulda, Dendrocalamus hamiltonii*, and *Stapletonia arunachalensis* all flowered in 2009 in Arunachal Pradesh, India. Subsequently, rodent outbreaks were reported in the flowering area, which caused severe damage to many crops (Kumawat et al., 2014). Consequently, people have begun to pay more attention to bamboo flowering for its scientific importance and its crucial role in many human communities.

All seed plants have similar life cycles, from seed germination, through a juvenile stage, vegetative growth phase, and reproductive period, including flowering and formation of seeds. Bamboo is similar, although it has its own unique flowering characteristics, which include: (i) it has a long vegetative phase; and (ii) its asexual reproductive capability is particularly strong, with a single clone possessing the ability to populate an entire bamboo forest. In addition, bamboo flowering has been linked to a number of poorly understood phenomena, such as bamboo groves bursting into bloom and dying, and sporadic flowering.

From more than 2,000 years ago to 1,721, many ancient Chinese books have recorded the phenomena of bamboo flowering and fruiting. However, most of these records mainly focus on the culinary and medicinal uses of bamboo fruit. The book *Shan Hai Jing*, written more than 2,000 years ago, states: “When bamboo flowers, it will wither. “The book *Zhu Pu*, written by Dai during the Jin Dynasty (from 317 to 420) states: “Bamboo flowering and seeding needs 60 years and bamboo can regenerate through seed in 6 years.” In 1,721, a local biography of Taizhou in Zhejiang province described the fruit of *Fargesia* sp., which could be used to treat dysentery (Zhou and Hu, 2000). From the mid-eighteenth to the early nineteenth century, researchers began to classify bamboo plants, but there were no reports of bamboo flowering. In fact, the first bamboo classification originates from Rumpf (1750), who divided the bamboos into eight classes, all with the name *Arundo*. Based on this division, in 1,753, Linnaeus used the name “*Arundo bambos*,” which included all the bamboos. Later, the genus name *Bambusa* was adopted (Holttum, 1956). In 1788, Retzius first established the genus of sympodial bamboo, using the name *Bambusa* (Jiang, 2007). In 1803, the first monopodial bamboo genus, *Arundinaria* Michaux, was established (Chao and Tang, 1993).

From 1,829 to the mid- nineteenth century, researchers paid closer attention to the reproductive organs, such as inflorescences and fruit, of bamboo plants. The earliest description of a bamboo inflorescence as a distinct structural unit was by Nees in 1829. This was quoted verbatim by Munro as a part of his characterization of the “Bambusaceae” (Munro, 1868; McClure, 1966). From the mid- nineteenth century to the early twentieth century, the amount of literature regarding bamboo flowering began to increase, and many reports used reproductive organs, such as inflorescences and fruits, as the main basis for classifying bamboo. For example, Munro used fruit characteristics as the main classification criterion, although the number of fruiting specimens was limited (Munro, 1868).

In the twentieth century, research on bamboo flowering greatly increased and inflorescence characteristics were widely used in the classification of bamboo. In addition, researchers comprehensively studied flowering-related events in bamboo, including flowering cycle, flowering habits, factors resulting in flowering, die-back and recovery, rejuvenation, and the effects of bamboo flowering (Lu, 1980; He et al., 1994; Chen et al., 1995; Cheng et al., 2014). In addition, McClure (1966) and Keng (1986) performed many detailed studies on inflorescences and the evolution of bamboo plants, and applied inflorescence characteristics to bamboo classification. Yao and Tan (2008) found that the fruits of only 72 species (out of 515 bamboo species) were described in the ninth volume of *Chinese Flora*. Since the beginning of the twenty-first century, studies on bamboo flowering have mainly focused on flowering biology (Qin, 1995; Zeng et al., 1998; Xing et al., 2005; Wang and Wu, 2009), molecular biology (Zhang et al., 2012; Gao et al., 2014, 2015; Wang et al., 2014), and the spatial and temporal distribution of flowering bamboo (Bhattacharya et al., 2006; Lin and Ding, 2007; Franklin, 2010; Zhang et al., 2012; de Carvalho et al., 2013; Gao et al., 2014; Wang et al., 2014; Crone et al., 2015).

Although bamboo flowering has been studied, many questions remain. Why do many sympodial bamboo species recorded in the literature often bloom sporadically? Why are differences in the flowering types of the same bamboo species observed in different areas? Why do the same bamboo species bloom in some areas and not in other areas? In this review, we address these questions by (I) summarizing and synthesizing information from many studies on the flowering cycle of bamboo, including more than 600 bamboo flowering events and 30 specific bamboo flowering events observed by researchers from the Bamboo Research Institute of Nanjing Forestry University. (II) Detailing the four types of bamboo flowering habits and summarizing the specific flowering cycles of 85 bamboo species. (III) Introducing a theory of the bamboo flowering cycle, which shows that each bamboo species and each clone has its own flowering cycle. In addition, we also introduce the flowering distribution and flowering wave phenomenon in bamboo, which explains why there are various flowering patterns in bamboo forests, and the reasons for bamboo flowering. (IV) Introducing two ways of rejuvenating flowering bamboo forests. We simplify the complicated flowering phenomena of various bamboo forests in nature using the bamboo flowering cycle theory to rationalize flowering phenomena.

## Types of Bamboo Flowering

To classify the types of bamboo flowering, we first collected as many examples of bamboo flowering as we could find. Researchers at the Bamboo Research Institute of Nanjing Forestry University have observed and studied the flowering events of many bamboo species. Table 1 lists the flowering events of 30 species of bamboo, including details on the flowering time, site, and type. We observed that all the flowering culms died after flowering in many species such as *Bambusa multiplex, Chimonobambusa sichuanensis, Phyllostachys edulis, Ph. glauca, Ph. nidularia*, and *Sasaella kogasensis* “Aureostriatus.” There are also some differences among different flowering bamboo species, mainly in the seed setting rate and the mode of bamboo forest regeneration and rejuvenation. It is worth noting that the observed flowering habits of some bamboo species were not completely consistent with the historical literature, which may be related to a change in environment and/or whether they were wild or cultivated.

**Table 1:** Bamboo flowering events observed by the Bamboo Research Institute of Nanjing Forestry University.

**Bamboo species**	**Flowering site**	**Flowering time**	**Literature reference**	**Flowering type**
*Bambusa multiplex*	Bamboo garden of Nanjing Forestry University, Jiangsu Province, China	2009–2016	(Lin et al., 2015)	Sporadic flowering; bamboo culm died after flowering
*B. oldhamii* “Xia Zao”	Xiapu County, Fujian Province, China	2013	(Lin et al., 2018)	Sporadic flowering; bamboo culm died after flowering
*Chimonobambusa sichuanensis*	Bamboo garden of Nanjing Forestry University, Jiangsu Province, China	2006–	(Lin et al., 2009)	Sporadic flowering; bamboo died after flowering; produced fruit
*Ch. utilis*	Bamboo garden of Nanjing Forestry University, Jiangsu Province, China	1986–1989	(Zhou, 1984)	Massive synchronized flowering; bamboo died after flowering; produced fruit
*Indocalamus tessellatus*	Bamboo garden of Nanjing Forestry University, Jiangsu Province, China	1994	(Mao and Zhang, 1996)	Massive synchronized flowering; bamboo died after flowering
*Indosasa patens*	Bamboo garden of Nanjing Forestry University, Jiangsu Province, China	1989, 1995	(Mao and Zhang, 1996)	Sporadic flowering; bamboo died after flowering
*Phyllosasa tranquillans* f. *shiroshima*	Bamboo garden of Nanjing Forestry University, Jiangsu Province, China	1996	(Mao and Zhang, 1996)	Partial flowering
*Phyllostachys arcana* f. *luteosulcata*	Bamboo garden of Nanjing Forestry University, Jiangsu Province, China	2007	(Lin and Ding, 2007)	Sporadic flowering; bamboo died after flowering; produced fruit
*Ph. edulis*	Guilin city, Guangxi Province, China	1970, 2005–2019	(Zhou, 1984) Observed by authors	Partial flowering; bamboo died after flowering; produced fruit
*Ph. fimbriligula*	Bamboo garden of Nanjing Forestry University, Jiangsu Province, China	1996	(Mao and Zhang, 1996)	Massive synchronized flowering
*Ph. glauca*	Jinan city and Linqing city, Shandong Province, China	2014	(Yue et al., 2018)	Massive synchronized flowering; bamboo died after flowering; produced fruit
*Ph. heteroclada*	Bamboo garden of Nanjing Forestry University, Jiangsu Province, China	2018	Observed by authors	Massive synchronized flowering; partial flowering bamboo plants withered; no seed after flowering
*Ph. heteroclada*	Bamboo garden of Nanjing Forestry University, Jiangsu Province, China	1987–1989	(Zhou, 1984)	Partial flowering; bamboo died; no seed after flowering
*Ph. nidularia*	Bamboo garden of Nanjing Forestry University, Jiangsu Province, China	1984	(Zhou, 1984)	Frequent sporadic flowering; flowering bamboo plants survived; no seed after flowering
*Ph. reticulata* f. *shouzhu*	Bamboo garden of Nanjing Forestry University, Jiangsu Province, China	1991	(Mao and Zhang, 1996)	Massive synchronized flowering; bamboo died after flowering
*Ph. rubromarginata*	Bamboo garden of Nanjing Forestry University, Jiangsu Province, China	1993	(Mao and Zhang, 1996)	Massive synchronized flowering; bamboo died after flowering
*Ph. violascens* f. *notata*	Bamboo garden of Nanjing Forestry University, Jiangsu Province, China	2007	(Lin and Ding, 2007)	Sporadic flowering; bamboo died after flowering
*Ph. viridiglaucescens*	Bamboo garden of Nanjing Forestry University, Jiangsu Province, China	2007	(Lin and Ding, 2007)	Sporadic flowering; bamboo died after flowering; produced fruit
*Ph. vivax*	Ge garden, Yangzhou city, Jiangsu Province, China	2015	(Zheng et al., 2016)	Sporadic flowering; bamboo died after flowering; produced fruit
*Pleioblastus argenteostriatus*	Bamboo garden of Nanjing Forestry University, Jiangsu Province, China	Since 1987	(Mao and Zhang, 1996)	Frequent sporadic flowering; bamboo died after flowering
*Pl. argenteostriatus* f. *angustitolius*	Taiping Zhen, Huangshan city, Anhui province, China	2015	(Zheng et al., 2016)	Sporadic flowering; bamboo died after flowering; produced fruit
*Pl. pygmaeus*	Bamboo garden of Nanjing Forestry University, Jiangsu Province and Sheyang, Yancheng City, Jiangsu Province, China	2015–2017	(Zheng et al., 2016)	Massive synchronized flowering; bamboo died after flowering; produced fruit
*Pl. simonii* f. *heterophyllus*	Bamboo garden of Nanjing Forestry University, Jiangsu Province, China	2007–2011	(Lin and Ding, 2013)	Sporadic flowering; bamboo died after flowering; produced fruit
*Pl. yixingensis*	Bamboo garden of Nanjing Forestry University, Jiangsu Province, China	2017	(Zhang et al., 2018)	Massive synchronized flowering; bamboo died after flowering; produced fruit
*Pseudosasa amabilis* var. *convexa*	Bamboo garden of Nanjing Forestry University, Jiangsu Province, China	Around 2002	(Zhang et al., 2003)	Sporadic flowering; bamboo died after flowering; produced fruit
*Sasaella kogasensis* ‘Aureostriatus’	Bamboo garden of Nanjing Forestry University, Jiangsu Province, China	2015–2017	(Zheng et al., 2016)	Massive synchronized flowering and fruiting; died after flowering
*Sasamorpha sinica*	Huangshan Scenic spot, Anhui Province, China	1951, 2001–2003	Observed by authors	Massive synchronized flowering and fruiting
*Semiarundinaria sinica*	Bamboo garden of Nanjing Forestry University, Jiangsu Province, China	2006–	Observed by authors	Sporadic flowering; no seed after flowering
*Shibataea chinensis*	Bamboo garden of Nanjing Forestry University, Jiangsu Province, China	2002–2010	(Lin and Ding, 2012)	Massive synchronized flowering; no seed after flowering; regeneration by asexual rejuvenation
*Sinobambusa tootsik*	Bamboo garden of Nanjing Forestry University, Jiangsu Province, China	2010	(Zheng et al., 2016)	Massive synchronized flowering and fruiting

*Due to the complexity of bamboo classification, the scientific names listed are all the scientific names in the original text, which may have changed since publication*.

Based on the extensive examples collected, we found that the flowering habits of bamboo can be divided into four types: sporadic, massive synchronized, combined massive synchronized and sporadic, and partial flowering (McClure, 1966; Lin and Mao, 2007). The details are shown in Table 2. Classifying the flowering phenomenon is done here according to the proportion of bamboo flowering in bamboo forests, hoping to unify thereby the classification criteria.

**Table 2:** Fairly reliable records of bamboo flowering habit.

**Flowering habit**	**Bamboo species**
Sporadic flowering	*Bambusa burmanica, B. distegia, B. emeiensis, B. intermedia, B. multiplex, B. oldhamii, B. remotiflora, B. rigida, B. spinosa, B. ventricosa, B. vulgaris, B. xiashanensis, B. xueana, Chimonobambusa ningnanica, Chimonocalamus pallens, Dendrocalamus barbatus, D. barbatus* var. *internodiradicatus, D. birmanicus, D. brandisii, D. fugongensis, D. giganteus, D. latiflorus, D. manipureanus, D. minor, D. pachystachyus, D. peculiaris, D. sinicus, D. yunnanicus, Drepanostachyum falcatum* var. *glomerata, Fargesia dracocephala, F. edulis, F. frigidis, F. hygrophila, F. melanostachys, F. papyrifera, Indosasa patens, I. sinica, Neomicrocalamus prainii, Phyllostachys angusta, Ph. arcana, Ph. bissetii, Ph. nidularia, Ph. praecox* “Viridisulcata,” *Ph. violascens, Ph. violascens* f. *preveynalis, Pleioblastus gramineus, Pl. simonii* f. *heterophyllus, Pseudosasa amabilis* var. *convexa, Ps. amabilis* var. *tenuis, Pseudostachyum polymorphum, Schizostachyum funghomii, Semiarundinaria densiflora* var. *villosum, Yushania sp*.
Massive synchronized flowering	*Acidosasa purpurea, Ampelocalamus patellaris, A. scandens, A. stoloniformis, Bambusa arnhemica, B. bambos, Cephalostachyum chinense, Ce. latifolium, Ce. pingbianense, Chimonobambusa pachystachys, Ch. quadrangularis, Ch. rigidula, Ch. szechuanensis, Ch. tumidissinoda, Ch. utilis, Chimonocalamus dumosus, Chusquea abietifolia, Ch. quila, Ch. ramosissima, Dendrocalamus longispathus, Fargesia denudata, F. fungosa, F. murielae, F. nitida, F. obliqua, F. qinlingensis, F. robusta, F. scabrida, F. spathacea, Gaoligongshania megalothyrsa, Gigantochloa albociliata, G. nigrociliata, Guadua trinii, Indocalamus tessellatus, I. wilsoni, I. angustata, Melocalamus compactiflorus, M. scandens, Otatea ramirezii, Phyllostachy atrovaginata, Ph. edulis, Ph. fimbriligula, Ph. glabrata, Ph. glauca* f. *Yunzhu, Ph. glauca, Ph. heteroclada* f. *solide, Ph. meyeri, Ph. nigra* var. *henonis, Ph. propinqua, Ph. rutila, Ph. sulphurea* var. *viridis, Ph. vivax, Pleioblastus amarus, Pl. argenteostriatus, Pl. linearis, Pl. maculatus, Pl. pygmaeus, Pseudosasa japonica, Sarocalamus faberi, Sasa kurilensis, S. palmata, S. senanensis, S. veitchii* var. *hirsuta, Sasaella kogasensis* ‘Aureostriatus’*, Sasamorpha sinica, Schizostachyum dumetorum, Sch. pergracile, Shibataea chinensis, Sinobambusa tootsik, Yushania confusa*
Combined massive synchronized and sporadic flowering	*Acidosasa notata, Bashania fargesii, Bambusa tulda, Chimonobambusa pachystachys, Chusquea ramosissima, Ch. culeou, Ch. macrostachya, Ch. montana, Ch. uliginosa, Ch. valdiviensis, Dendrocalamus hamiltonii, D. hamiltonii* var. *hamiltonii, D. membranaceus, D. strictus, Indocalamus latifolius, Melocanna baccifera, Phyllostachys heteroclada, Ph. reticulata, Ph. reticulata* f. *shouzhu, Ph. reticulata* f. *tanakae, Ph. rubromarginata, Sasa cernua, Schizostachyum dullooa*
Partial flowering	*Acidosasa notata, Indocalamus* sp., *Phyllosasa tranquillans* f. *shiroshima, Ph. iridescens, Ph. reticulata* f. *lacrima-deae, Pleioblastus amarus* var. *pendulifolius, Pl. simonii*

*Due to the complexity of bamboo classification, the scientific names listed are all the scientific names in the original text, which may have changed*.

In sporadic flowering, there are usually only 1–2 clusters or a small area of scattered bamboo flowering within a bamboo population. This flowering occurs randomly. As shown in Table 2, 53 bamboo species are listed as undergoing sporadic flowering, including common species such as *Bambusa emeiensis, Dendrocalamus latiflorus*, and *Ph. violascens*. According to the literature (Zhou, 1984; Du et al., 2000; Yuan et al., 2005, 2008, 2012; Franklin, 2010), sporadic flowering often occurs in cultivated or intensively managed bamboo species, which flower sporadically more often than wild species. Franklin (2010) suggested that the term “sporadic flowering” may imply random or other non-gregarious patterns of flowering, but application of the term has been variable and ill-defined, and there is no convincing evidence that any semelparous bamboo has a reproductive strategy that may be regarded as “not gregarious.” This paper holds that sporadic flowering is a random and irregular small number of flowering.

In massive synchronized flowering, also known as gregarious flowering, a large flowering area of >50% occurs within a bamboo population. Many bamboo plants show a cyclic pattern of gregarious flowering after a long vegetative period (Janzen, 1976; Du et al., 2000). As shown in Table 2, 70 bamboo species undergo massive synchronized flowering. Many researchers are concerned about this flowering type, because massive synchronized flowering of bamboo forests leads to large-scale bamboo death, which seriously affects the local economy and environment.

In the combined massive synchronized and sporadic flowering category, species may display sporadic and/or small areas of flowering before and after large areas undergo flowering. As shown in Table 2, 23 bamboo species, such as *B. tulda, Chusquea culeou, C. montana, Melocanna baccifera, Ph. heteroclada, Ph. reticulata*, and *Sasa cernua*, are in this category (Zhang W. Y. et al., 1992; Pearson et al., 1994; Du et al., 2000; Ramanayake and Weerawardene, 2003; Bhattacharya et al., 2006; Kitamura and Kawahara, 2009; Wang and Wu, 2009; Tagle et al., 2013).

In partial flowering, the degree of flowering in a bamboo forest is between sporadic and massive synchronized flowering and generally occurs in a patchy distribution. There are seven bamboo species listed as partial flowering in Table 2. For example, *Pleioblastus simonii* underwent partial flowering at Kew Gardens from 1892 to 1903 (Bean, 1907).

We investigated whether there is a relationship between the flowering habits of bamboo species and their taxonomic position at the genus level. We found that some genera, such as *Arundinaria, Bambusa, Chimonobambusa, Dendrocalamus, Phyllostachys, Pleioblastus*, and *Schizostachyum*, included sporadic, massive synchronized, and combined massive synchronized and sporadic flowering species (Bean, 1907; Jiao, 1956; Anonymous, 1961; Ram and Gopal, 1981; Zhou, 1984; Zhang and Ma, 1989; Pearson et al., 1994; Du et al., 2000; Li and Denich, 2004; Bhattacharya et al., 2006; Mao et al., 2008; Kitamura and Kawahara, 2009; Nath and Das, 2010; Sarma et al., 2010; Xu et al., 2012, 2014; Tagle et al., 2013; Inoue et al., 2014; Xie et al., 2016; Zheng et al., 2016). Some bamboo genera, such as *Fargesia, Indosasa, Pseudosasa*, and *Yushania*, included sporadic and massive synchronized flowering species (Zhang and Ma, 1989; Du et al., 2000; Li and Denich, 2004; Jiang, 2007). In some genera, such as *Acidosasa, Ampelocalamus, Cephalostachyum, Drepanostachyum, Gaoligongshania, Gigantochloa, Melocalamus, Sasaella, Shibataea*, and *Sinobambusa*, only massive synchronized flowering bamboo species have been observed (Du et al., 2000; Ramanayake and Weerawardene, 2003; Kumawat et al., 2014; Zheng et al., 2016). However, some bamboo genera, such as *Neomicrocalamus, Pseudostachyum*, and *Semiarundinaria*, contain only sporadic flowering species (Zhang and Ma, 1989; Du et al., 2000; Yuan et al., 2005). Obviously, there are some genera which only show one model, while others show more. This issue is more complicated and may be related to the number of species in the genus or the distribution range of species. Generally speaking, the genera with a large number of species are more widely distributed, and different flowering types are more likely to be seen. However, the differences of flowering types in different species within the same genus are mainly determined by the biological characteristics of the species. For example, it is a very common phenomenon that some species in *Phyllostachys* blossom sporadically, such as *Phyllostachys nidularia*, but some species usually show massive synchronized flowering, such as *Ph. glauca, Ph. reticulata*. Another example is that several flowering types can be observed in a bamboo species. *Ph. heteroclada*, a bamboo species widely distributed in China, massively bloomed in 1958 in Shennongjia, Hubei Province, China, and partially bloomed in 1987–1989 in Nanjing Forest University, China. In addition, it bloomed massively in Yaan, Sichuan Province, China in 2003–2007, and flowered sporadically in 1995 in Yiliang, Yunnan Province, China (Zhou, 1984; Du et al., 2000; Li and Denich, 2004; Wang and Wu, 2009). Therefore, bamboo flowering type can only be determined according to the specific flowering behavior of a bamboo population. There is no obvious relationship between the flowering habit of bamboo species and the taxonomic position at the genus level.

Bamboo flowering type is closely correlated with whether the bamboo forest is wild or cultivated. Du et al. (2000) studied the flowering phenomena and types of 61 bamboo species belonging to 23 genera in Yunnan, China. They divided bamboo flowering into massive synchronized and sporadic flowering, and showed that the flowering and fruiting characteristics of bamboo species were closely related to their status as wild or cultivated, as well as with their taxonomic relationship at the genus level.

## The Flowering Cycle of Bamboo

The period between two gregarious flowerings is generally called the flowering cycle, and the flowering cycles of different bamboo species are variable. The flowering cycle of bamboo ranged from 3 to 150 years (Kurz, 1876; Janzen, 1976) (Table 3). Brandis (1899) divided bamboo flowering habits into three types: annual, periodic, and uncertain.

**Table 3:** Records of flowering cycle of some bamboo species.

**Species**	**Flowering cycle (years)**	**References**
*Ampelocalamus scandens*	29	(Zhang J. X. et al., 1992)
*Acidosasa notata*	>50	(Xu et al., 2012)
*Bambusa arnhemica*	41–51	(Franklin, 2010)
*B. balcooa*	32–34	(Fan and Qiu, 1987)
*B. bambos*	30–40, 47–52	(Kurz, 1876; Bourdillon, 1895; Nicholls, 1895; Brandis, 1899, 1906; Troup, 1921; Blatter and Parker, 1929; Blatter, 1930; Dutra, 1938; Ueda, 1960; Mitra and Nayak, 1972; Janzen, 1976; Fu, 1985; Bennet et al., 1990; Tewari, 1993; Seethalakshmi and Muktesh Kumar, 1998; Poudyal, 2009)
*B. copelandii*	47–48	(Raizada, 1948; McClure, 1966)
*B. nutans* subsp. *cupulata*	35	(Poudyal, 2009)
*B. polymorpha*	54–60, >50, >68, 80	(Kwe, 1904; Bradley, 1914; Seifriz, 1923; Jiao, 1956; Ueda, 1960; Janzen, 1976)
*B. teres*	35–60	(Fu, 1985; Fan and Qiu, 1987)
*B. tulda*	48	(Ram and Gopal, 1981)
*B. vulgaris*	150+	(Janzen, 1976)
*Bashania fargesii*	50–60	(Liu and Fu, 2007)
*Chimonobambusa quadrangularis*	100+	(McClure, 1966)
*Ch. utilis*	~60	(Zhang et al., 1994)
*Chusquea abietifolia*	30–34	(Seifriz, 1920, 1950; Janzen, 1976)
*Ch. culeou*	12, 14–20, 61–62	(Janzen, 1976; Pearson et al., 1994; González and Donoso, 1999; Jaksic and Lima, 2010; Tagle et al., 2013; Guerreiro, 2014)
*Ch. lorentziana*	32	(Guerreiro, 2014)
*Ch. montana*	41	(Guerreiro, 2014)
*Ch. quila*	15–20, 45	(Janzen, 1976; Guerreiro, 2014)
*Ch. ramosissima*	23, 29	(Dutra, 1938; Guerreiro, 2014)
*Ch. tenella*	15–16	(Dutra, 1938; Guerreiro, 2014)
*Ch. valdiviensis*	50–70	(González and Donoso, 1999; Tagle et al., 2013)
*Dendrocalamus faleatun*	28–30	(Janzen, 1976; Zhou, 1984; Lin et al., 2009)
*D. giganteus*	40, ~76	(Janzen, 1976; Lin, 2009)
*D. hamiltonii*	25, 30, 44	(Gupta, 1972; Janzen, 1976; Fan and Qiu, 1987)
*D. hookeri*	117	(Janzen, 1976)
*D. strictus*	8–9, 12–15, 20–70, 7–70	(Troup, 1921; Kadambi, 1949; Jiao, 1956; Ueda, 1960; McClure, 1966; Shah, 1968; Wang and Chen, 1971; Gupta, 1972; Khan, 1972; Janzen, 1976; Fu, 1985; Fan and Qiu, 1987; Wu, 1988)
*Drepanostachyum falcatum*	20–30, 35	(Lowndes, 1947; Jiao, 1956; Fu, 1985; Fan and Qiu, 1987; Lin, 2009)
*D. intermedium*	10	(Brandis, 1899)
*Fargesia denudata*	50–60, 63	(Shao, 1986; Yu et al., 1987; Liu and Fu, 2007)
*F. murielae*	35, 80–100, 110	(Tredici, 1998; Li and Denich, 2004)
*F. nitida*	50–60	(Qin, 1995)
*F. robusta*	50–60	(Liu and Fu, 2007)
*F. scabrida*	50–60	(Qin, 1995; Liu and Fu, 2007)
*F. spathacea*	35, 110	(Li and Denich, 2004)
*Guadua chacoensis*	31	(Guerreiro, 2014)
*G. paraguayana*	38	(Guerreiro, 2014)
*G. sarcocarpa*	26–29	(Nelson, 1994; de Carvalho et al., 2013)
*G. trinii*	30–32	(Dutra, 1938; Janzen, 1976; Guerreiro, 2014)
*G. weberbaueri*	27–28	(Guerreiro, 2014)
*Himalayacalamus falconeri*	20–38	(Tingle, 1904; Bean, 1907; Holttum, 1956; Janzen, 1976; Fan and Qiu, 1987)
*Indocalamus latifolius*	~100	(Anonymous, 1961)
*I. tessellatus*	60, >115	(McClure, 1966; Yuan et al., 2008)
*Kuruna wightiana*	1	(Janzen, 1976; Fan and Qiu, 1987)
*Melocanna baccifera*	7–10, 26–50	(Ueda, 1960; Janzen, 1976; Ram and Gopal, 1981; Fu, 1985; Fan and Qiu, 1987; Lin et al., 2009; Singleton et al., 2010; Govindan et al., 2016)
*Merostachys anomala*	30	(Dutra, 1938)
*M. burchellii*	30	(Dutra, 1938)
*M. clausenii*	32	(Guerreiro, 2014)
*M. fistulosa*	30–34	(Janzen, 1976; Jaksic and Lima, 2010)
*M. multiramea*	31–33	(Budke et al., 2010; Guerreiro, 2014)
*M. skvortzovii*	30–34	(Guerreiro, 2014)
*M. sp*.	11	(Janzen, 1976)
*Nastus elegantissimus*	3	(Kurz, 1876)
*Neehouzeaua dullooa*	14–17	(Gupta, 1972)
*Neololeba amahussana*	1	(Fan and Qiu, 1987)
*Ochlandra scriptoria*	1	(McClure, 1966)
*O. stridula*	1	(Janzen, 1976; Zhou, 1984; Lin et al., 2009)
*O. travancorica*	7, 28–30	(Broun, 1887; Jiao, 1956; Janzen, 1976; Fu, 1985; Fan and Qiu, 1987)
*Oldeania alpina*	~40	(Janzen, 1976)
*Otatea acuminata* subsp. *aztecorum*	30–35	(Anonymous, 2004)
*O. ramirezii*	8–30	(Ruiz-Sanchez, 2013)
*Oxytenanthera abyssinica*	7–21	(Fanshawe, 1972; Janzen, 1976)
*Phyllostachys aurea*	13–19	(Janzen, 1976)
*Ph. dulcis*	42–43	(Adamson, 1978)
*Ph. edulis*	>48, 67	(Janzen, 1976; Watanabe et al., 1980)
*Ph. fimbriligula*	>60	(Chen et al., 1995)
*Ph. glauca*	50–60 or 120	(Zhang, 1977)
*Ph. heteroclada*	50–60, ~80	(Li and Denich, 2004; Wang and Wu, 2009)
*Ph. nigra*	40–50	(Lin et al., 2010)
*Ph. nigra* var. *henonis*	40–50, 58–63	(Kawamura, 1927; Wu, 1988; Li and Denich, 2004; Lin, 2009)
*Ph. reticulata*	40–50, 60 or 100, 115, 120, >100	(Kawamura, 1927; Ueda, 1960; Numata, 1970; Chen, 1973; Janzen, 1976; Lu, 1980; Zhou, 1984; Li and Denich, 2004)
*Pleioblastus argenteastriatus*	>26	(Lin et al., 2017)
*Pl. pygmaeus*	>26	(Lin et al., 2017)
*Pl. simonii* var. *variegata*	30	(McClure, 1966)
*Rhipidocladum neumannii*	21	(Guerreiro, 2014)
*Sarocalamus faberi*	44–55	(Yu et al., 1987; Qin et al., 1989)
*S. racemosus*	30–31	(Jiao, 1956; Janzen, 1976)
*Sasa kurilensis*	>100	(Inoue et al., 2014)
*Sasaella kogasensis* ‘Aureostriatus’	>31	(Lin et al., 2017)
*Schizostachyum dullooa*	37–48	(González et al., 2002; Giordano et al., 2009; Marchesini et al., 2010; Nath and Das, 2010)
*Sch. pingbianense*	46	(Du et al., 2000)
*Thamnocalamus spathiflorus*	10–11, 16–17	(Brandis, 1899; Janzen, 1976)
*Thyrsostachys oliveri*	48	(McClure, 1966)
*Yushania confusa*	88	(Li and Denich, 2004)
*Yushania maling*	50+	(Ray, 1952)

*Due to the complexity of bamboo classification, the scientific names listed are all the scientific names in the original text, which may have changed*.

In this review, we compiled more than 600 records of bamboo flowering events of 85 bamboo species from the literature, of which 187 had a defined flowering cycle. As shown in Table 3, some species that belong to apparently iteroparous bamboos, which grow to maturity and then flower and seed annually for many years, were termed annual flowering. This included *Neololeba amahussana, Ochlandra scriptoria, O. stridula*, and *Kuruna wightiana* (McClure, 1966; Janzen, 1976; Zhou, 1984; Fan and Qiu, 1987; Lin, 2009). However, the term annual flowering bamboo species in the literature remains to be discussed because it will take several years for some bamboo species from initial flowering to the end of flowering. For example, *Pseudosasa amabilis* blooms for 15 years, so it is easily mistaken for an annual flowering bamboo species (Lin and Mao, 2007). It is important to distinguish, in perennial bamboo species, annual flowering bamboo species from bamboos whose flowering periods last for a long time.

We found that the flowering cycles of different bamboo species, even those belonging to the same genus, fluctuate greatly. For example, the flowering cycle of *Arundinaria* varied from 10 to 60 years, *Bambusa* from 30 to 150+ years, *Chusquea* from 12 to 70 years, and *Phyllostachys* from 13 to 120 years (Brandis, 1899; Kawamura, 1927; Ueda, 1960; Numata, 1970; Chen, 1973; Janzen, 1976; Zhang, 1977; Lu, 1980; Zhou, 1984; Fu, 1985; Pearson et al., 1994; González and Donoso, 1999; Li and Denich, 2004; Liu and Fu, 2007; Jaksic and Lima, 2010; Tagle et al., 2013; Guerreiro, 2014). The flowering cycle in *Guadua* was less variable and relatively stable, being between 26 and 33 years (Dutra, 1938; Janzen, 1976; Nelson, 1994; de Carvalho et al., 2013; Guerreiro, 2014). In addition, for the same bamboo species, the flowering cycle observed by different researchers in different locations varied. For example, *B. bambos* flowered in 1804, 1836, 1868, and 1899 in Brazil, giving it a flowering cycle of 31–32 years (Dutra, 1938). In Dehra Dun, Uttar Pradesh, India, it flowered in 1836, 1881, and 1926, resulting in a flowering cycle of 45 years (Blatter and Parker, 1929). In Upper Weinganga Valley, Balaghat District, India, it flowered in 1818 and 1865–1870, giving it a flowering cycle of 47–52 years (Nicholls, 1895).

Janzen (1976) described the flowering cycles of 41 species of bamboo in detail. Guerreiro (2014) surveyed and estimated the flowering cycles of 16 woody bamboo species in Argentina and neighboring areas. Franklin (2010) recorded and summarized the flowering cycle of *B. arnhemica*. Their work revealed that the flowering cycle of the same bamboo species is not fixed and can be quite different. For example, the flowering cycle of *Ph. edulis* was generally believed to be 67 years (Watanabe et al., 1980), but according to other records, *Ph. edulis* has not flowered in Fenghua, Zhejiang Province, China for more than 200 years (Chai et al., 2006). This phenomenon may be caused by differences in flowering cycles in different clonal bamboo forests or the influence of environmental factors, especially those of managed plantations.

The synchronous flowering phenomenon of bamboo, which has attracted much attention, is unique. Janzen (1976) proposed a predator escape hypothesis to explain the synchronous flowering of bamboo. This hypothesis assumes that seed predators will eat up the seeds when bamboo forests blossom sporadically. Reproduction by seeds is only ensured when sufficient amounts of seeds are produced to overcome the loss via seed predators. Crone et al. (2015) also revealed that synchronous reproduction among conspecifics has several demonstrated fitness benefits, including enhanced rates of pollination, increased attraction of seed dispersers, and reduced seed predation. Veller et al. (2015) proposed a simple mathematical model that supported a two-step evolutionary process: first, an initial phase in which a mostly annual flowering population synchronizes to a small multi-year interval; and second, a phase of successive small multiplications of the initial synchronization interval, resulting in the extraordinary intervals seen today. Other researchers believe that many species have general synchronous flowering tendencies over large continental regions. Campbell (1985) noted a remarkable concentration of mean periods with multiples of 15–16 years among 20 taxa, most of them from southeastern Asia. Guerreiro (2014) showed that several species of woody bamboo native to southern South America undergo synchronous flowering, with flowering cycles of ~30 years.

Researchers calculate the flowering cycle by subtracting two flowering times. This is only accurate if the later flowering bamboo is the offspring of the previously flowering bamboo. Thus, observing at least three generations is required for accurate prediction of a flowering cycle. However, the present data on bamboo flowering may be questionable. First, it is very easy to find data based on only a single recorded generation of flowering cycle. Second, several different flowering events are described in many bamboo species, but it is not known whether the bamboo forests in a flowering event were derived from the seeds produced from the last flowering event. Finally, the historical records of bamboo flowering are limited. For many bamboo species, only one mass flowering event has been recorded, but the historical flowering time of the bamboo forest and the subsequent seed flow are not described in detail. Although there is not a definitive way to address these issues, the historical data still provide an important reference.

## Bamboo Flowering Cycle Theory

Based on the comprehensive historical documents and the field observations of the researchers at the Bamboo Research Institute of Nanjing Forestry University over the past decades, some conclusions about the bamboo flowering cycle theory are proposed and summarized.

First, a bamboo species has a certain flowering cycle, or rather, each ramet (that is, individuals with the same genotype from the same fertilized egg) of bamboo has a certain flowering cycle. In addition, differences in flowering cycles among different bamboo species are greater than those between different clones of the same species. For example, *Phyllostachys glauca* bloomed in 1909 to the west of Zhejiang, China, and partially bloomed in 1965 in Fuyang District, Zhejiang Province, China. In addition, *Ph. glauca* bloomed massively in Jinlu Village of Yinzhou District, Zhejiang Province, China since 1990, and also flowered in 2013–2016 in Linqing and Jinan Districts, Shandong Province, China (Anonymous, 1972; Xu and Chen, 1992). However, *Ph. glauca* did not blossom in other places, such as Nanjing, Jiangsu Province, China. This indicates that the *Ph. glauca* plants in these places were probably derived from different clones, with different specific flowering times and flowering cycles.

Clonal bamboo has very similar flowering cycles. For example, *Sasaella kogasensis* “Aureostriatus” was introduced from the Fuji Bamboo Garden in Japan to Nanjing Forestry University of China in 1984 by Professor Zhou. Subsequently, the bamboo was introduced from Nanjing Forestry University in all parts of China. Since 2015, forests of *S. kogasensis* “Aureostriatus” have been blooming one after another in China, indicating that the same clone keeps flowering synchronously. Nevertheless, changes in the local environment may result in slightly different flowering cycles. Thus, although the flowering cycle of the same clonal bamboo is theoretically stable, it is affected by the environment. If the clonal bamboo is susceptible to disturbances, then its flowering time will vary greatly in different environments. However, if clonal bamboo is not easily disturbed, then the flowering time is less varied. For example, *Ph. glauca* growing in the Royal Botanical Gardens of England and Japan, which were introduced from the west of Zhejiang, China, bloomed in 1907 (Cheng et al., 2014).

Second, flowering period may last several years in a bamboo forest and it generally goes through three phases: small areas of sporadic flowering, massive synchronized flowering, and small areas of sporadic flowering. This phenomenon is called the “bamboo flowering distribution”. Synchronous flowering refers to a flowering distribution in which the vast majority of clumps within a patch initiate flowering in a given year, with most of the remaining flowering the year before or after (Abe and Shibata, 2012). Suyama et al. (2010) proposed that sporadic flowering may occur as a result of mechanistic malfunction, and result in massive flowering, therefore this should be regarded as part of the normal massive-flowering schedule. For example, we observed that *Pleioblastus pygmaeus* began sporadic flowering in 2015 in the Bamboo Garden of Nanjing Forestry University in Jiangsu Province, China. By 2016, the number of flowering bamboo stems peaked at ~85%, but a few flowering clumps occurred until 2019. The proportion of clumps that flower during the peak period varies among different bamboo species, such as 80–90% for *Sarocalamus faberi* (Taylor and Qin, 1988), 95.7% for *B. arnhemica* (Franklin, 2010), and 96.5% for *C. culeou* (Marchesini et al., 2010).

Third, gregarious flowering occurs in patches over successive years, and this has been described as a “flowering wave.” These waves have been widely reported among bamboo species and are repeated in successive generations (Franklin, 2010). Franklin (2010) hypothesized that bamboo flowering waves were the product of environmentally induced increments in the flowering schedules in which the underlying periodicity was reset at germination by an endogenous biological clock. When part of a population is subjected to such an increment, and that part is sufficiently aggregated in time and space to maintain the viability derived from synchronicity, an offset (patch) is established that will be maintained across generations. Flowering waves are always temporally organized but are not necessarily spatially organized (Franklin, 2010; Abe and Shibata, 2012). This phenomenon represents the interaction of endogenous and exogenous factors. Bamboo is a special plant with a long vegetative growth period. Bud mutations occur frequently in bamboo plants, including *Ph. edulis, Ph. edulis* f. *pachyloen, B. multiplex* and its variant *B. multiplex* var. *riviereorum*. Bamboo forests are more likely to mutate during the period of sexual reproduction (Janzen, 1976; Franklin, 2010; Hanlon et al., 2019). Therefore, even if the plants in a bamboo forest all come from the same clone at the beginning, it is possible that there will be variation within the forest. In addition, the environment affects bamboo flowering. Generally, internal and external factors lead to different flowering cycles and form the flowering wave.

In accordance with the bamboo flowering references, four types of bamboo flowering have been summarized (Lin and Mao, 2007). Why are various flowering types observed? The reasons for these phenomena are as follows: On the one hand, the flowering events in bamboo forests generally form temporally structured but spatially chaotic flowering distributions, which undergo three phases: small areas of sporadic flowering, massive synchronized flowering, and small areas of sporadic flowering. Therefore, different flowering types can be obtained by observing the bamboo forest during different periods. On the other hand, it also depends on whether the bamboo forest is populated by the same clonal bamboo. The flowering cycles of different ramets of bamboo are different. Therefore, if the bamboo forest is composed of the same clone, then it will form a flowering distribution and complete the reproductive phase. However, if the bamboo forest is composed of different clones, then there will be different flowering times for the different clones. Thus, when one of the clones begins flowering, the other clones may still be in the vegetative stage. As a result, it is possible to form flowering waves, and researchers often observe sporadic flowering or partial flowering in a bamboo forest. Thus, a variety of flowering patterns are often observed in bamboo forests.

In the past, the causes of bamboo flowering remained controversial. Many theories have emerged, including growth cycle, nutrition, external cause, free radicals, pathology, individual variation, mutation, periodic aging, and rejuvenation (Janzen, 1976; Sharma, 1994; Keeley and Bond, 1999; Chai et al., 2006; Wang, 2013). Kawamura (1927) proposed that periodicity in bamboo must be the product of an endogenous mechanism that was relatively immune to environmental influences. Franklin (2010) offered hypotheses on the interactions of endogenous and exogenous cues to flowering that may lead to the development of flowering waves and other flowering patterns in bamboo. He also suggested that stressors on the plant might override or force the clock, such as triggering hormone production independently of the clock. It is possible that the threshold of physiological stress required to induce flowering decreases as the scheduled time of flowering approaches (Franklin, 2010). Recently, researchers have reached a consensus on the cause of bamboo flowering. They believe that bamboo flowering is modulated by environmental conditions, but the root cause is internal genetic factors and flowering occurs when bamboo grows to its physiologically mature age. External factors, such as climate and human disturbance, can advance, delay, or stop the occurrences of bamboo flowering events to a certain extent (Franklin, 2010). For example, the flowering time of bamboo can be delayed or advanced to a certain extent by applying exogenous hormones (Ding, 2007). However, the specific key factor that induces the initiation of flowering in bamboo is still unknown. We also hypothesize that endogenous hormones, which tend to be signaling molecules, may be key factors for initiation, but this requires further study.

In a word, the flowering cycle of bamboo is very important for predicting future bamboo flowering times. Janzen (1976), Franklin (2010), and Guerreiro (2014) have presented summaries of the flowering cycle of bamboo. Ma et al. (2017) found a negative correlation between the rate of molecular evolution and flowering cycle in Arundinarieae. Bamboo species with longer flowering cycles tend to evolve more slowly than those with shorter flowering cycles (Montti et al., 2011).

## Rejuvenation and Regeneration of Flowering Bamboo Forests

Regeneration of a robust bamboo forest has major economic and ecological implications. There are generally two types of bamboo forest rejuvenation after flowering: sexual and asexual. The clonal composition of a bamboo forest will be changed after flowering and natural regeneration.

### Regeneration Through Sexual Reproduction

Some bamboo species bloom and produce large amounts of seeds, which fall to the ground and germinate into seedlings. Seeds can grow into seedlings, and each seedling represents a clone. The flowering process of bamboo forests can last from several to more than 10 years, so the seedlings produced by the seeds may be of different ages. The bamboo species *Chimonobambusa utilis* (Zhang, 1985), *Ch. pachystachys* (Mao et al., 2008), *Ch. tumidissinoda* (Dong et al., 2001), *Fargesia denudata* (Shao, 1986), *F. nitida* (Yu et al., 1987), *Melocanna arundina* (Chai et al., 2006), *Phyllostachys edulis* (Qin, 2015), and *Sarocalamus faberi* (Qin et al., 1989; Taylor and Qin, 1993) belong to this type. *S. faberi* seedlings take ~15–20 years to develop into mature stands after a bamboo flowering event (Taylor and Qin, 1993). Thus, after flowering and rejuvenation, more clones can be formed in the bamboo forest.

### Regeneration Through Asexual Reproduction

Here, the bamboo forest is rejuvenated by the flowering bamboos rhizomes and the buds of culm bases. In a flowering bamboo forest, the buds on the rhizomes of the flowering bamboo can sprout and form dwarf and weak bamboo, which usually bloom in the same year and will grow new rhizomes underground. In the following years, the buds of the new rhizomes sprout and form dwarf and weak bamboos that coexist with flowers and leaves. Thus, the flowering bamboo forest can form normal non-flowering bamboo after several years (Xiong et al., 1979). During the process of asexual rejuvenation, the proportion of flowering bamboo generally first rises and then falls, while the proportion of non-flowering bamboo falls and then rises. At the end, the bamboo forest does not blossom at all. Some bamboo species renew their forests in this way, such as *Pleioblastus amarus* (Zheng and Huang, 1990), *Pl. amarus* var. *pendulifolius* (Zheng and Huang, 1990), *Phyllostachys reticulata* (Lu, 1980), *Ph. atrovaginata* (He et al., 1994), *Ph. vivax* (Xiong et al., 1979), *Ph. praecox* f. *prevernalis* (Chai et al., 2006), *Ph. fimbriligula* (Chai et al., 2006), and *Shibataea chinensis* (Lin and Ding, 2012).

The process of regeneration and rejuvenation of this type of flowering bamboo forest is slow. For example, *Ph. reticulata* requires 7–10 years (Lu, 1980), *Ph. vivax* requires 6–7 years (Xiong et al., 1979), and *Pl. amarus* and *Pl. amarus* var. *pendulifolius* require at least 10 years (Zheng and Huang, 1990). However, artificial measures can be used to improve the growth conditions of bamboo forests and accelerate regeneration. For example, it only took 5–6 years for *Ph. atrovaginata* to recover to the pre-flowering production level under intense management conditions (He et al., 1994). During the process of asexual rejuvenation, variation may also occur. For example, *Pleioblastus pygmaeus* flowered during the period from 2015 to 2018 in Baima Bamboo Resource Nursery of Nanjing Forestry University (119°07′42″E, 31°37′55″ N), Nanjing, Jiangsu Province, China. After flowering the authors of this paper observed that many bamboo clumps of *Pl. pygmaeus* with white-striped leaves appeared in the bamboo forest. Therefore, the clonal composition of a bamboo forest after asexual rejuvenation may not be identical to that of the bamboo forest before flowering.

There are three bamboo fruiting types that are characterized based on seed production: those that produce a large number of seeds, those that rarely produce seeds, and those having no seeds. A few bamboo species have no seeds and can only regenerate their forests through asexual rejuvenation. For example, Lin and Ding (2012) observed the flowering of *Shibataea chinensis* at Nanjing Forestry University from 2002 to 2010, and found it had no seeds. Some bamboo species have a low seed setting rate, such as *Bambusa emeiensis, B. multiplex*, and *Ph. heteroclada*, and some have high seed setting rate, such as *Ph. edulis, Ph. vivax, Pl. pygmaeus*, and *Sasaella kogasensis* “Aureostriatus.” Generally speaking, most bamboo species with few seeds or a large number of seeds regenerate their forests through sexual reproduction and asexual rejuvenation after flowering. For example, *B. multiplex, Indocalamus tessellatus, Ph. glauca, Pl. pygmaeus*, and *S. kogasensis* “Aureostriatus” can be renewed in either manner after flowering (Cheng et al., 2014). There may be a preferred approach, but the specific selection of the regeneration manner may be correlated with the growth environment. In addition, a few species can only be propagated through sexual reproduction, such as *Fargesia murielae*.

## Explanations of Natural Phenomena Using the Bamboo Flowering Cycle Theory

The bamboo flowering cycle theory described in this review may explain many strange and complex phenomena associated with bamboo flowering.

Genetically similar bamboo of a range of different ages coexist in space and time, forming a continuous spectrum of clones. This age structure may eventually lead to sporadic or partial flowering in the same bamboo forest, with no obvious regularity in flowering.

The following examples illustrate the continuous spectrum of clones. First, we take *Phyllostachys edulis*, a scattered bamboo species, as an example. *Ph. edulis*, distributed in Guilin, Guangxi Province, China began to bloom and bear fruits in a large area in 1963, and from 1995 until the present it had small areas of continuous blooming every year, where a significant amount of breeding was carried out (Li and Gu, 2003). Because flowering time does not occur at the same time for a whole bamboo forest, and it probably maybe last many years, the age of the offspring produced by it will also be different, therefore, a continuous spectrum of clones has formed in this bamboo forest. When a certain clone reaches the flowering age, the other clones may be still in vegetative growth. So when this flowering clone renews and resumes vegetative growth, other clones may reach the flowering age and start blooming. Consequently, the bamboo forest will show continuous patchy flowering for many years. The other example is *Chimonobambusa utilis*, a species distributed in Tongzi, Guizhou Province, China. Due to many clones of different ages, bamboo forests of *Ch. utilis* have had patchy flowering phenomenon every year since 2006. Third, according to historical documents some bamboo species frequently blossom sporadically, such as *Bambusa distegia, B. emeiensis, B. intermedia, B. oldhamii, B. multiplex*, and *Dendrocalamus latiflorus*. These bamboo species belong to sympodial bamboos, in their bamboo forests, they form a series of clones. In the same time and space, there are bamboos of different ages in the bamboo forest, so they will bloom in different years (Supplementary Video 1).

## Conclusion and Perspectives

Bamboo plays an important role in human life, especially in countries that have rich bamboo resources. Bamboo flowering and the subsequent death of entire forests results in huge economic losses and environmental problems. Therefore, it is necessary to understand the flowering cycle of economically important bamboo species. Bamboo flowering is a normal biological phenomenon, the transformation from vegetative to reproductive growth. It is the last stage of ontogeny and is an inevitable process of a biological organism's development (Wang, 2013).

The historical data that have been recorded lack many key details. Owing to the characteristic long lifespan of bamboo, it is very difficult to observe its full cycle in a generation (Guerreiro, 2014). The flowering cycle must be obtained by relying on the flowering events recorded in successive generations. In addition, with the advancement of civilization, many wild bamboo populations have disappeared, making it nearly impossible to observe flowering events in their original place. Consequently, this review suggests that seeds should be collected and seeded after the flowering and fruiting of bamboo plants. The seedlings can be separately planted in scientific research institutions that study bamboo, where they can be marked and managed. Similarly, data on bamboo forests that undergo asexual regeneration should be recorded. In this way, the next flowering times of bamboos in different regions, the variations of flowering in different clones, and the effects of the environment on bamboo flowering can be recorded.

Additionally, a database similar to the web-based Flora of China should be established. This would allow experts and non-experts to contribute to our understanding of bamboo flowering. It is necessary to record bamboo flowering events in detail, including information on the origin and whereabouts of the bamboo. At the same time, the identification of bamboo plants is difficult and error-prone. Therefore, it is essential to encourage scientific exchanges and validation. Finally, the website should contain information about the bamboo industry, technology, and other related aspects, so that it can become a useful resource for the bamboo industry.

The long flowering period of bamboo has long been a fascinating mystery. However, by examining historical data and generating accurate data going forward, we have begun to understand the flowering of this important and remarkable plant. Exploration of the molecular mechanisms setting the flowering time for bamboo, using knowledge leveraged from model plants, particularly other monocots, remains an exciting field for future research.

## Author Contributions

XZ wrote the manuscript. YD and SL critically reviewed and added to the review. HF and YW contributions to acquisition of data, or analysis of data. All authors read and commented on this manuscript.

## Conflict of Interest

The authors declare that the research was conducted in the absence of any commercial or financial relationships that could be construed as a potential conflict of interest.

## Supplementary Material

Supplementary Video 1 Dynamic demonstation of flowering wave.
